# Application of Near Infrared Reflectance Spectroscopy for Rapid and Non-Destructive Discrimination of Hulled Barley, Naked Barley, and Wheat Contaminated with *Fusarium*

**DOI:** 10.3390/s18010113

**Published:** 2018-01-02

**Authors:** Jongguk Lim, Giyoung Kim, Changyeun Mo, Kyoungmin Oh, Geonseob Kim, Hyeonheui Ham, Seongmin Kim, Moon S. Kim

**Affiliations:** 1 Department of Agricultural Engineering, National Institute of Agricultural Sciences, Rural Development Administration, 310 Nongsaengmyeng-ro, Wansan-gu, Jeonju 54875, Korea; limjg@korea.kr (J.L.); giyoung@korea.kr (Gi.K.); cymoh100@korea.kr (C.M.); yoonmine@korea.kr (K.O.); kgs1733@naver.com (Ge.K.); 2 Microbial Safety Team, National Institute of Agricultural Sciences, Rural Development Administration, 166 Nongsaengmyeong-ro, Iseo-myeon, Wanju-gun 55365, Korea; hhham@korea.kr; 3 Department of Bioindustrial Machinery Engineering, College of Agriculture & Life Sciences, Chonbuk National University, 567 Baekje-daero, deokjin-gu, Jeonju 54896, Korea; 4 Environmental Microbial and Food Safety Laboratory, Agricultural Research Service, US Department of Agriculture, 10300 Baltimore Avenue, Beltsville 20705, MD, USA; Moon.Kim@ars.usda.gov

**Keywords:** *Fusarium*, near infrared, discrimination, hulled barely, naked barley, wheat

## Abstract

*Fusarium* is a common fungal disease in grains that reduces the yield of barley and wheat. In this study, a near infrared reflectance spectroscopic technique was used with a statistical prediction model to rapidly and non-destructively discriminate grain samples contaminated with *Fusarium*. Reflectance spectra were acquired from hulled barley, naked barley, and wheat samples contaminated with *Fusarium* using near infrared reflectance (NIR) spectroscopy with a wavelength range of 1175–2170 nm. After measurement, the samples were cultured in a medium to discriminate contaminated samples. A partial least square discrimination analysis (PLS-DA) prediction model was developed using the acquired reflectance spectra and the culture results. The correct classification rate (CCR) of *Fusarium* for the hulled barley, naked barley, and wheat samples developed using raw spectra was 98% or higher. The accuracy of discrimination prediction improved when second and third-order derivative pretreatments were applied. The grains contaminated with *Fusarium* could be rapidly discriminated using spectroscopy technology and a PLS-DA discrimination model, and the potential of the non-destructive discrimination method could be verified.

## 1. Introduction

Barley (*Hordeum vulgare* L.) is one of the four major grains, and is the next most important crop after rice in South Korea. It is now mainly used for mixed rice, barley tea for children, and various organic food materials [1]. Barley has many functional nutrients such as β-glucan, arabinoxylan, and hydrocyanic acid, which other general grains do not have, and has effects that aid in the prevention of adult diseases, such as heart disease (by lowering the blood cholesterols) and obesity (by inhibiting body fat accumulation) [2,3]. Barleys can be generally classified into hulled barley, which is not easily peeled and is used as a feed, and naked barley, which is easily peeled and can be used for food [4]. Recently, as consumers’ interest in health has increased, hulled barley has been actively developed into processed products such as barley tea, malt, and barley sprouts [5,6]. Wheat (*Triticum aestivum* L.) is second only to corn in world grain production, and is rich in nutrients such as saccharides, proteins, and vitamins. However, most of the wheats consumed in Korea depend on imports. The wheats produced domestically are used for producing bread, noodles, and pasta, and we are trying to increase the self-sufficiency of Korea’s wheat production [7,8].

*Fusarium* (particularly *F. graminearum*) occurs worldwide in Asia, Canada, Europe, and South America [9]. In Korea, *Fusarium* occurred on a large scale in the southern part of the country in 1963, and the production of barleys decreased by 40–80%, which became a big social issue [10]. *Fusarium* is one of the most destructive plant pathogens; it reduces crop yields through causing red rot fungus in barley and wheat, ear and stem-rotting disease in corn, and root-rotting disease in many other plants [11]. The grains contaminated with *Fusarium* turn into empty grains and decrease in weight, thus reducing yields and causing enormous economic losses [12]. In particular, the secondary metabolite mycotoxin, which is produced by such molds, is generated during the growing, storage, and distribution of contaminated agricultural products, since it is stable to heat and does not disappear, even when heated. Thus, when contaminated grains are ingested with normal grains by people and livestock, mycotoxins can accumulate in the body and cause serious acute and chronic disorders. Above all, mycotoxin is classified as a carcinogen in nature [13].

Quantification methods using high performance liquid chromatography are often used for the inspection of grain contamination by *Fusarium* and mycotoxin in research and official analysis methods. For the contamination of *Fusarium*, the main method involves cultivating the spores that appear as fungi growth and checking them with the naked eye. For the discrimination of mycotoxin, liquid chromatography [14], gas chromatography (GC) [15,16], GC with mass spectrometry [17], and enzyme-linked immunosorbent assay (ELISA) [18,19] are used. These culture and chemical analysis methods are highly sensitive and accurate. However, they require the destruction of the samples, experts who can handle expensive analysis equipment, and are limited in rapid discrimination because it takes a long time [20]. Meanwhile, near infrared reflectance spectroscopy is being used as a technique for quickly and non-destructively discriminate grains contaminated with *Fusarium*. 

Near infrared reflectance spectroscopy (NIRS) is an analytical method that uses a 700–2500 nm wavelength band based on the absorption of near infrared rays by organic compounds and water. More than 60 years have passed since the first practical application of this technique. Karl Norris, the pioneer of NIRS, first attempted this technique in the 1960s to measure the water content of grains and seeds [21].

Since then, instrumentation, statistical methods, and software have improved, and application techniques have increased exponentially in many different fields. Now, the American Association of Cereal Chemists (AACC 39-00) and the American Oil Chemist Association (AOCS am 1–9) have approved NIRS as an analysis method for grains and seeds. The NIR techniques have several important advantages, such as short analysis time, minimal sampling process, and non-destructiveness with a performance comparable to chemistry analytical methods. There are many calibration algorithms for spectra acquired from NIRS, which share the same principles with and are similar to the methods that are widely used in quantitative analysis, such as multiple linear regression (MLR), principal component regression (PCR), and partial least squares (PLS) [22].

PLS and PCR show very similar results, and MLR is more advantageous when handling areas with short wavelengths or data points that have little correlations. In particular, PLS is used as a statistical modeling tool for obtaining correlations between multivariate input variables (independent variables) and output variables (dependent variables) using data acquired from experiments [23]. It is actively used to develop linear multivariate model in diverse industrial fields, including chemical process [24,25,26]. This method offers the advantage of a model with a higher prediction reliability than general multiple regression analysis, even if there is multicollinearity or a lot of noise in the data, because the input variables have high correlations with one another. The partial least square discrimination analysis (PLS-DA) is a supervised classification method used in NIRS, because it allows near infrared reflectance (NIR) variables (wavelengths) with high correlations.

This study was conducted to develop a technology to discriminate hulled barley, naked barley, and wheat contaminated with *Fusarium* using a near infrared spectroscopy measurement system. For this purpose, a PLS-DA model was developed by applying various mathematical pretreatments to a reflectance spectrum acquired from a spectrometer with a wavelength range of 1175–2170 nm, and its prediction performance was verified through correct classification rate (CCR).

## 2. Materials and Methods 

### 2.1. Hulled Barley, Naked Barley, and Wheat Samples

**Figure 1:**
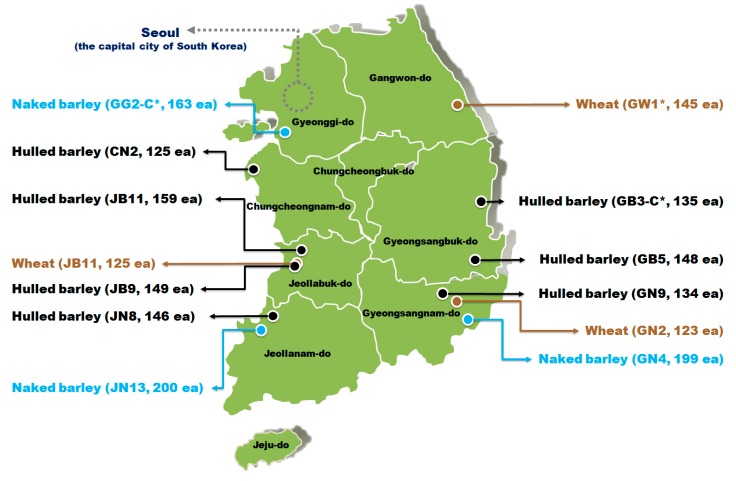
Geographic locations and numbers of hulled barley, naked barley, and wheat used in the experiments from each location. Sample groups marked with an asterisk (*) are control groups.

The hulled barley, naked barley, and wheat samples in this study used grains harvested from various regions in 2015, as shown in Figure 1. Hulled barley samples were collected from seven locations, including three locations in Gyeongsangbuk-do, one location in Gyeongsangnam-do, two locations in Jeollabuk-do, one location in Jeollanam-do, and two locations in Chungcheongnam-do. Naked barley samples were collected from one location each in Gyeonggi-do, Jeollanam-do, and Gyeongsangnam-do. Wheat samples were collected from one location each in Gangwon-do, Jeollabuk-do, and Gyeongsangnam-do. Each sample was kept separately in an individual container at a low temperature of 4 °C to prevent the multiplication of *Fusarium*. The hulled barley, naked barley, and wheat samples in which no *Fusarium* was found in the first culture experiment were used as the control group.

As shown in Table 1, the hulled barley samples of the GB3-C group from the Gyeongsangbuk-do region were used as the control group, because no samples contaminated with *Fusarium* were found in the first culture result. The hulled barley samples collected from the other six locations were used as the experimental group. For the naked barley samples, the GG2-C group from the Gyeonggi-do region were used as the control group, and the samples collected from the other two locations were used as the experimental group. For wheat samples, the GW1-C group from the Gangwon-do region were used as the control group, because no wheat contaminated with *Fusarium* was found, and the samples collected from the other two locations were used as the experimental group. The hulled barley, naked barley, and wheat samples used in this experiment were assigned individual sample numbers. As shown in Table 1, the grain samples were placed separately in a tray with 10 × 10 indented cells in the shape of grains.

### 2.2. NIRS Measurement System

**Figure 2:**
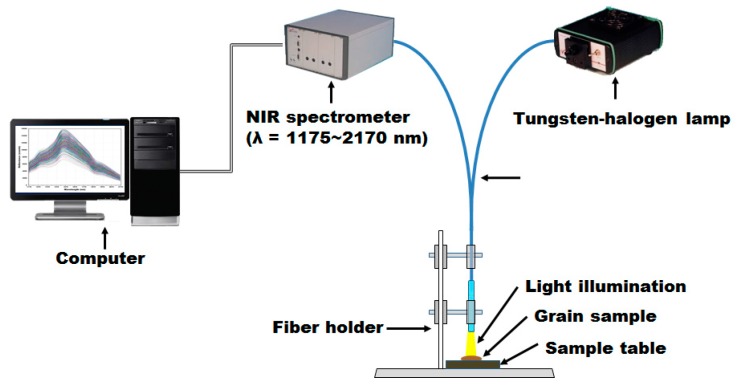
A schematic diagram of the near infrared reflectance spectroscopy (NIRS) measurement system for the discrimination of *Fusarium* contamination of cereals.

As shown in Figure 2, the NIRS measurement system is comprised of a NIR spectroscopic sensor, a tungsten-halogen lamp, a fiber optic probe, a sample table, a fiber holder, and a measurement computer.

The NIR spectroscopic sensor (Avaspec-NIR256-2.2TC, Avantes BV Inc., Apeldoorn, The Netherlands) has a wavelength range of 1175–2170 nm, a pixel pitch of 3.4 nm, 256 pixels in total with a pixel size of 50 × 500 μm, and an indium gallium arsenide (InGaAs) linear array sensor. For the tungsten-halogen lamp used to supply light in the NIR wavelength range, a light source with 10 W power (Avalight-HAL, Avantes BV Inc., Apeldoorn, The Netherlands) and a wavelength range of 360–2500 nm was used. The bifurcated fiber-optic probe (FCR-7IR400-2-ME, Avantes BV Inc., Apeldoorn, The Netherlands), which transmits light from the light source and receives the light reflected from the grain sample with the NIR spectrometer, consists of seven optic fibers with a diameter of 400 μm. Furthermore, the AVA soft program (Avasoft-basic, Avantes BV Inc., Apeldoorn, The Netherlands) was used to acquire and store the reflectance spectrum data from the grain samples.

### 2.3. NIR Reflectance Spectrum Acquisition

**Figure 3:**
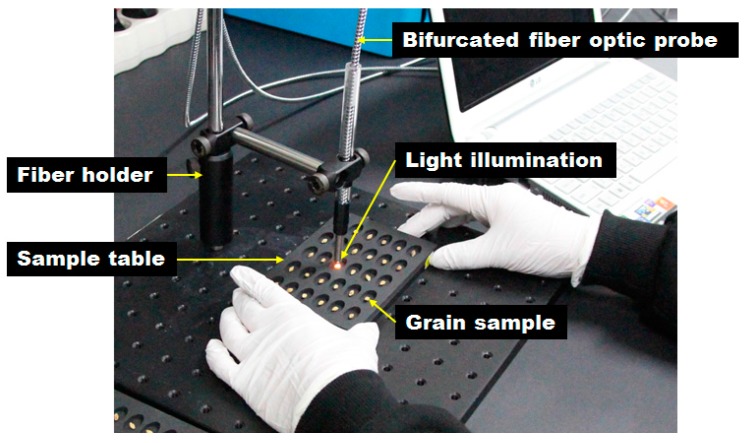
Bifurcated fiber-optic probe and light illumination in near infrared reflectance (NIR) spectrum acquisition for hulled barley, naked barley, and wheat.

To acquire the NIR reflectance spectra of hulled barley, naked barley, and wheat, the grains were moved to the dedicated template for measurement, as shown in Figure 3. A total of six measurements were made for each grain sample to acquire six NIR reflectance spectra. The distance between the grain sample and the end of the fiber-optic probe was maintained at 10–15 mm, and the light from the light source was irradiated to the surface of the grain samples as much as possible. A total of 5976 reflectance spectra were acquired from 996 hulled barley samples, a total of 3372 reflectance spectra were acquired from 562 naked barley samples, and a total of 2358 spectra were acquired from 393 wheat samples. The measurement condition was 200 ms of light integration time, and the cumulative average was obtained from three measurements. In addition, smoothing pretreatment was applied by default for the acquisition of reflectance spectrum.

### 2.4. Culturing Fusarium

After hulled barley, naked barley, and wheat were measured for the acquisition of the NIR reflectance spectrum, a culture experiment was performed to determine *Fusarium* contamination for each grain of the samples. First, the medium was prepared to promote the cultivation of *Fusarium* latent in the grains. For the medium, 23.4 g and 5.04 g of Difco^TM^ potato dextrose agar and Bacto^TM^ agar, respectively, in powder state were weighed and mixed. Then, 900 mL of distilled water was added to two types of mixed medium powders, and 225 mL each of the mixture was poured into each of four bottles. The four medium solutions in the bottles were sufficiently stirred with hotplate with magnetic stirrers (MSH-30A, DAIHAN Scientific Co., Ltd., Wonju, Korea) until they were fully dissolved. The prepared medium solutions were sterilized for 15 minutes at 120 °C with a high-pressure sterilizer (AC-1 60 L, Universal Scientific Co., Ltd., Gimpo, Korea) and left on a clean bench for about one hour to cool down to 40 °C. Two types of antibiotics were injected to the medium solutions to prevent the growth of germs other than *Fusarium*. For the first antibiotic, 0.5 g Neomycin was melted in 50 mL distilled water and filtered through a 0.2 μm filter; then, 10.8 mL of the filtered antibiotic was injected into the medium solution. For the second antibiotic, 2.5 g Streptomycin was melted in 50 mL distilled water and filtered through a 0.2 μm filter; then, 18 mL filtered antibiotic was injected to the 900 mL medium solution. The injected two antibiotics were sufficiently mixed by stirring for 30 s using the hotplate with magnetic stirrers. Half of the medium solution with antibiotics added was poured into a 125 × 125 mm square dish (SP11125, SPL Life Sciences Co., Ltd., Pocheon, Korea), which could hold 36 grains and had been gas-sterilized. The dish was left for 2–3 h to harden the medium. Then, 36 grains were placed on the medium in each prepared square dish using sterilized tweezers. The used tweezers were sterilized with an alcohol lamp and cooled off before using again. The square dish with 36 grains was covered and sealed with tape so that no germs would enter it. The sealed square dishes were cultured for 2–4 days in an incubator at 25 °C, and the final *Fusarium* spores were verified by visual inspection.

### 2.5. Development of Fusarium Discrimination Prediction Model

The prediction model used to discriminate the grains contaminated with *Fusarium* was PLS-DA. For the developed model, the CCRs (%) for normal and contaminated samples were calculated, respectively. Unlike the partial least square regression model—in which a linear regression model is developed with dependent variables—and spectra data—which is an independent variable—PLS-DA, through using the actually measured concentration data [27,28], develops a regression model by specifying the groups to be discriminated as dummy variables instead of using the concentration values as the dependent variable [29]. The independent variables used to develop the PLS-DA model were the NIR reflectance spectra acquired from the hulled barley, naked barley, and wheat samples. For the dependent variables, the spectra acquired from the normal hulled barley, naked barley, and wheat samples that were not contaminated with *Fusarium* was specified as ‘0’ as a random virtual variable, and the spectra acquired from the contaminated grain samples was specified as ‘1’. 

For the raw spectrum data, spectra pretreatment was applied by various mathematical methods to correct light scattering by irregular surface appearance and develop a good discrimination prediction model [30]. To correct the movement of the spectra baseline due to surrounding environmental conditions such as the changed lighting and temperature when measuring spectra, first derivative, second derivative, and third derivative pretreatments were applied. To remove the light scattering effect during spectrum measurement, mean scattering correction (MSC) and standard normal variate (SNV) were used. Cross-validation statistics were applied to estimate the predictive ability of the PLS-DA calibration models developed during this research. The accuracy of the developed models was determined by its coefficient of determination for calibration (RC2), the standard errors of calibration (SEC), the coefficient of determination for validation (RV2), the standard error of prediction (SEP), and the principal component (PC). Furthermore, the baseline method was applied to correct the minimum values of spectra identically. A multivariate data analysis software application (Unscrambler v9.2, Camo Co., Oslo, Norway) was used for the pretreatment of the acquired reflectance spectra, the development of the PLS-DA model with the converted spectra, and the correction and validation of the developed model.

## 3. Results and Discussion

### 3.1. Culture Results of Hulled Barley, Naked Barley and Wheat

Table 2 shows the culture results for hulled barley, naked barley, and wheat. The first culture results in column (a) show the results of preliminary culture experiment for samples, which were used to determine whether the samples collected from each region were a contaminated group or a normal group. In this study, the control group and experimental group of the hulled barley, naked barley, and wheat samples were classified by the first culture experiment results. The number of grain samples was identical to the number of grains used in the preliminary culture experiment. The second culture experiment was carried out after the near infrared reflectance spectrum was acquired, and the results are shown in column (b).

**Figure 4:**
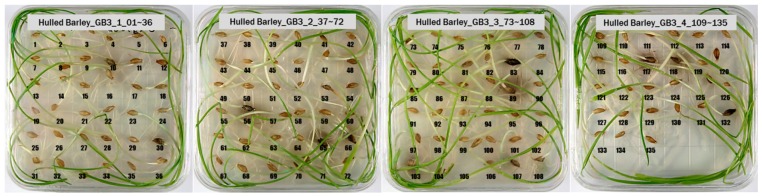
Culture results for hulled barley (GB3-C) in the control group, showing that the *Fusarium* spore was not observed.

For the hulled barley samples, a total of 996 grains including the GB3-C samples, which were the control group, and the samples collected from six regions, which were used as the experimental group, were placed in the square dishes—36 grains per dish—and were cultured. After four days, the cultured samples were discriminated by experts with the naked eye for contamination with *Fusarium*. In the control group (GB3-C), no spore contaminated with *Fusarium* was observed in the preliminary and second culture experiments. In the case of JB11, which was used as an experimental group, one grain was found to be contaminated with *Fusarium* in the preliminary culture experiment, but in the main experiment, no contaminated hulled barley grain was found. In the other experimental groups, *Fusarium* spores were observed, although the quantities were different. Therefore, 135 GB3 grains were used as the control group samples for discriminating hulled barley grains contaminated with *Fusarium*. For the experimental groups samples, a total of 150 grains were used, including GB5 (1 grain), GN9 (106 grains), JB9 (26 grains), JN8 (15 grains), and CN2 (2 grains) for hulled barley samples contaminated with *Fusarium*. Figure 4 shows the status of the GB3 samples after culture, which were the hulled barley samples that were used as the control group. Normal hulled barley germinated with no generation of spores.

**Figure 5:**
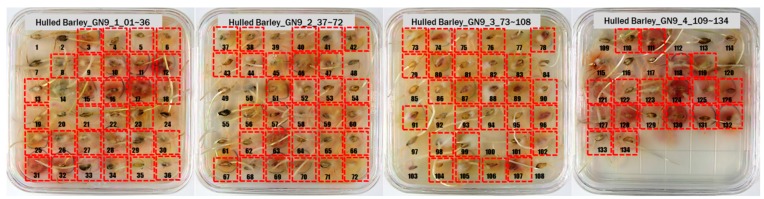
Culture results for hulled barley (GN9) in the experimental group, showing that the *Fusarium* spore was observed. The hulled barley samples that generated spores were marked by red squares.

Figure 5 shows the state after culture of the GN9 samples, in which 106 grains generated *Fusarium* spores: the largest number among the hulled barley sample groups that were used as experimental groups. The samples that generated spores were marked by red dotted lines.

**Figure 6:**
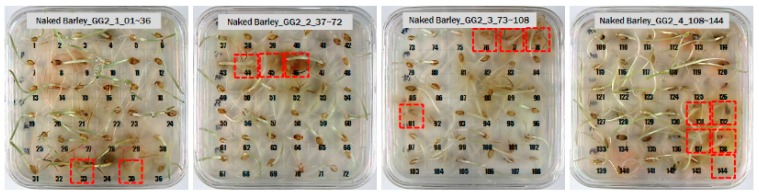
Culture results for naked barley (GG2-C) in the control group, showing that the *Fusarium* spore was observed. The naked barley samples that generated spores were marked by red squares.

For the naked barley samples, a total of 562 grains, including GG2-C samples, which were the control group, and the samples collected from the other two regions, which were used as the experimental groups, were placed and cultured in the square dishes. No *Fusarium* spores were found in the GG2-C sample group, which was the control group, in the preliminary culture experiment results. In the second culture experiment, however, 14 grains were contaminated with *Fusarium* among the 163 grains in total, as shown in Figure 6.

**Figure 7:**
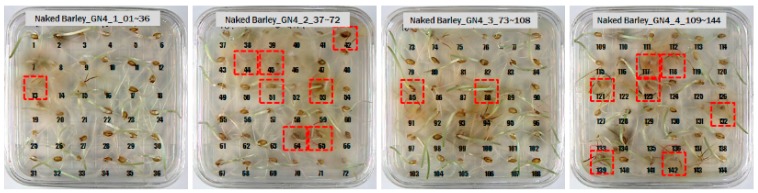
Culture results for naked barley (GN4) in the experimental group, showing that the *Fusarium* spore was observed. The naked barley samples that generated spores were marked by red squares.

Among the experimental groups, 12 out of the 200 grains in the JN13 sample group and 21 out of the 199 grains in the GN4 group were contaminated. Figure 7 shows the samples of the GN4 group, which were the naked barley samples that were used as the experimental group, in which *Fusarium* spores were generated. For the control group for discriminating naked barley samples contaminated with *Fusarium*, 149 grains that were not contaminated with *Fusarium* in the GG2-C group were used. For the experimental group, a total of 33 grains, including JN13 (12 grains) and GN4 (21 grains), were used.

**Figure 8:**
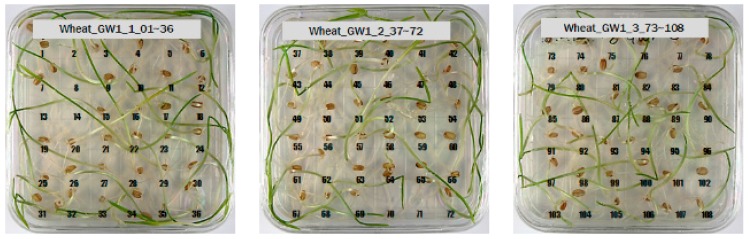
Culture results for wheat (GW1) in the control group, showing that the *Fusarium* spore was not observed.

For wheat samples, a total of 393 grains were placed and cultured in square dishes, including GW1-C samples of the control group, and the samples collected from the other two regions in the experimental groups. In the GW1-C wheat samples of the control group, no spores contaminated with *Fusarium* were found, and normal wheats were germinated, as the second culture experiment results show in Figure 8.

**Figure 9:**
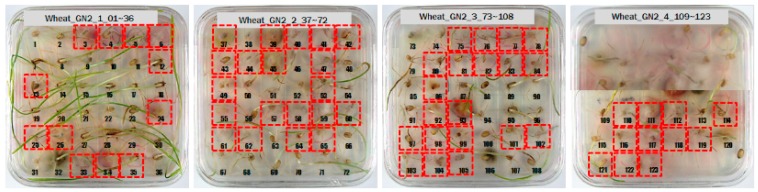
Culture results for wheat (GN2) in the control group, showing that the *Fusarium* spore was observed. The wheat samples that generated spores were marked by red squares.

Among the wheat samples in the experimental groups, a total of 96 grains were found to be contaminated with *Fusarium*, including 33 grains in the JB11 group, and 63 grains in the GN2 group. Figure 9 shows wheat samples of the GN2 group in which *Fusarium* spores were observed. 

### 3.2. NIR Reflectance Spectra of Hulled Barley, Naked Barley, and Wheat

**Figure 10:**
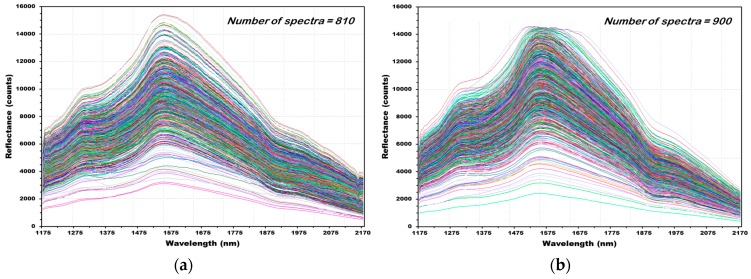
All of the NIR reflectance spectra of hulled barley samples not contaminated with *Fusarium* (NCF) (**a**) and contaminated *Fusarium* (CF) (**b**).

Figure 10 shows the reflectance spectra of 1710 (Not contaminated with *Fusarium* (NCF): 810, contained with *Fusarium* (CF): 900) obtained from 285 hulled barley samples (NCF: 135, CF: 150). Figure 10a shows the raw reflectance spectra acquired using NIR256 spectroscopic sensor for 135 grains in the hulled barley samples of the control group that were not contaminated with *Fusarium*. First, 810 reflectance spectra were collected by measuring 135 samples six times. The highest rising peak occurred at 1575 nm, and the strength of reflectance at this time was 15,500 counts at the maximum. A rising peak also occurred in the 1335 nm wavelength, and a falling peak occurred at the 1922 nm wavelength. Figure 10b shows the 900 NIR reflectance spectra acquired from 150 grains of hulled barley samples that were contaminated with *Fusarium*, which show a similar trend as the reflectance spectra of hulled barley samples. However, the second rising peak occurred at the 1327 nm wavelength, resulting in a shift.

**Figure 11:**
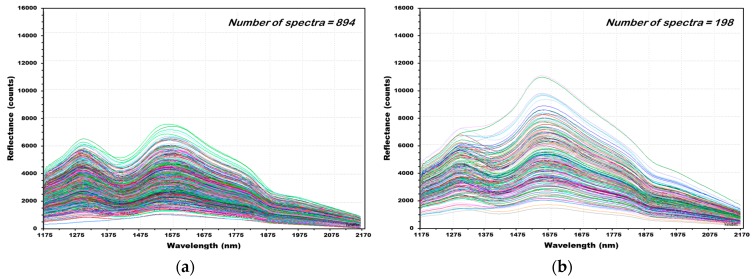
All NIR reflectance spectra of naked barley samples NCF (**a**) and CF (**b**).

Figure 11 shows the reflectance spectra of 1092 (NCF: 894, CF: 198) obtained from 182 naked barley samples (NCF: 149, CF: 33). Figure 11a shows 894 raw reflectance spectra acquired from 149 grains in total of the group naked barley samples in the control group that were not contaminated with *Fusarium*. Similar to the hulled barley, the maximum rising peak of the naked barley samples occurred at 1579 nm. However, the strength of the reflected light decreased, because the surface of naked barleys with no skin absorbed more light, and the maximum strength of reflectance was around 7500 counts, which is low. The second rising peak occurred at the 1309 nm wavelength, and the maximum strength of reflectance was 6500 counts. The falling peak occurred at 1933 nm. Figure 11b shows the 198 raw NIR reflectance spectra acquired from 33 naked barley grains contaminated with *Fusarium*. 

**Figure 12:**
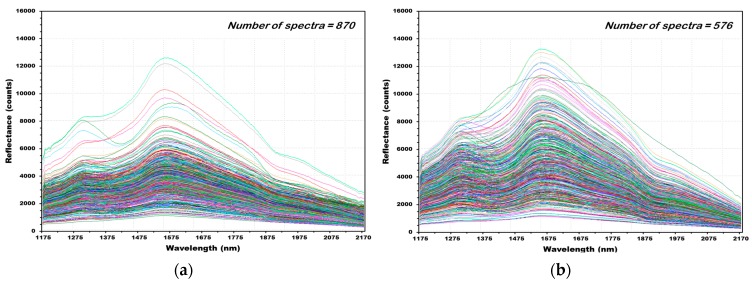
All NIR reflectance spectra of wheat samples NCF (**a**) and CF (**b**).

Figure 12 shows the reflectance spectra of 1446 (NCF: 870, CF: 576) obtained from 241 wheat samples (NCF: 145, CF: 96). Figure 12a shows 870 raw reflectance spectra acquired from 145 grains of wheat samples that were not contaminated with *Fusarium*. The maximum rising peak occurred at 1579 nm wavelength, where those of hulled barley and naked barley grains occurred. The second rising peak occurred at 1314 nm. Figure 12b shows 576 raw reflectance spectra acquired from 96 grains of wheat samples contaminated with *Fusarium*. The first and second rising peaks occurred at wavelengths similar to those of normal wheat.

### 3.3. Results of the PLS-DA Prediction Model for Fusarium Discrimination

A PLS-DA prediction model was developed to discriminate *Fusarium* contamination using the result of culturing *Fusarium* after acquiring near infrared reflectance spectrum from hulled barley, naked barley, and wheat samples. Table 3 shows the CCR result for the calibration and validation performance of the PLS-DA models developed by applying various mathematical pretreatments to the NIR reflectance spectra acquired from hulled barley, naked barley, and wheat samples. The CCR of the PLS-DA discrimination prediction model for hulled barley, naked barley, and wheat that has been developed with acquired raw reflectance spectra is 98% or higher, indicating good results. The CCR for the validation performance of the raw reflectance spectra to which no pretreatment was applied was 100% (810/810) for the NCF of hulled barley samples, and 99.44% (895/900) for the CF, with 99.72% on average. The CCR of the naked barley samples was 99.89% (893/894) for NCF and 99.48% (195/198) for CF, with 99.18% on average. The CCR of wheat samples was 99.66% (867/870) for NCF and 99.83% (576/576) for CF, with 99.74% on average, showing the best discrimination performance. For the mathematical pretreatments for the three types of grain samples, the derivative pretreatment showed the most appropriate and improved performance. In the validation performance of hulled barley samples, the discrimination prediction performance of the second derivative improved to R_V_^2^ = 0.946 and SEP = ±0.116. The CCRs of NCF and CF were 100% and 99.78% (898/900), respectively. For the validation performance of naked barley samples, all of the CCRs of NCF and CF in the first, second, and third derivatives improved to 100%. Thus, the spectra pretreatment that applied derivatives effectively improved the discrimination performance. In particular, the validation performance applying the third derivative pretreatment was R_V_^2^ = 0.948 and SEP = ±0.088, showing the best performance among all of the samples. In the wheat samples, too, the CCR of the spectra pretreatment applying derivative improved. The prediction model of the PLS-DA that developed by applying second derivative pretreatment showed the best results at R_V_^2^ = 0.933 and SEP = ±0.127.

#### 3.3.1. Prediction Results of the PLS-DA Model for Discrimination of Contaminated with *Fusarium* (CF)-Hulled Barley

**Figure 13:**
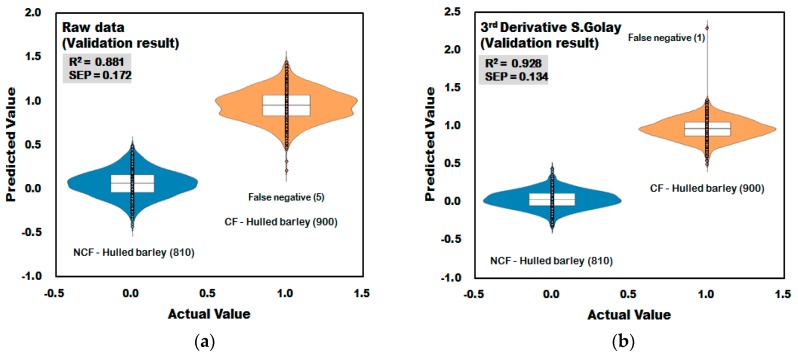
Validation results for *Fusarium* discrimination of the PLS-DA model developed using raw reflectance spectra (**a**) and the third-order derivative pretreatment (**b**) obtained from hulled barley.

Figure 13a shows the validation result of the PLS-DA model developed with no application of pretreatment to the raw reflectance spectra acquired from the hulled barley samples. In the raw data, five out of 900 spectra acquired from 150 grains of CF hulled barley were predicted as false positive at 0.5 or lower at the reference value of ±1, resulting in 99.44% (895/900) CCR. The 810 spectra from the hulled barley samples that were not contaminated showed 100% CCR. In this study, various mathematical spectra pretreatments were applied to improve the accuracy of the prediction model. As shown in Figure 13b, the third-order derivative result predicted only one of the 900 spectra acquired from CF as higher than 1.5, which is the reference value, resulting in a CCR of 99.89% (899/900). The CCR of the third-order derivative pretreatment was 100% (810/810) for NCF. The validation model applying the third-order derivative pretreatment showed R_V_^2^ = 0.928, which improved from 0.881, and is the result of the raw spectra in Figure 13a, and SEP decreased to ±0.134, proving the effect of spectra pretreatment.

#### 3.3.2. Prediction Result of PLS-DA Model for Discrimination of CF-Naked Barley

**Figure 14:**
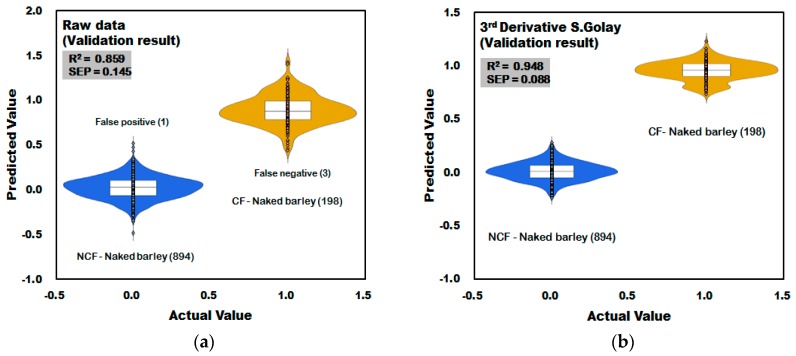
Validation results for *Fusarium* discrimination of the PLS-DA model developed using raw reflectance spectra (**a**) and the third-order derivative pretreatment (**b**) obtained from naked barley.

Figure 14a shows the result of the PLS-DA model developed using the raw spectra acquired from naked barley samples. In the raw data in Figure 14a, one of the 894 spectra acquired from NCF-naked barley samples was predicted as higher than 0.5, which is the reference value, resulting in a CCR of 99.89% (893/894). In the CF-naked barley samples, three of the 198 spectra was predicted as lower than 0.5, which is the reference value, resulting in a CCR of 98.48% (195/198). Figure 14b shows the PLS-DA model developed by applying third-order derivative pretreatment to the raw spectra acquired from naked barley samples. Validation performance of NCF-naked barley and CF-naked barley showed 100% CCR. The validation model improved to R_V_^2^ = 0.948 and decreased to SEP = ±0.088.

#### 3.3.3. Prediction Result of PLS-DA Model for Discrimination of CF-Wheat

**Figure 15:**
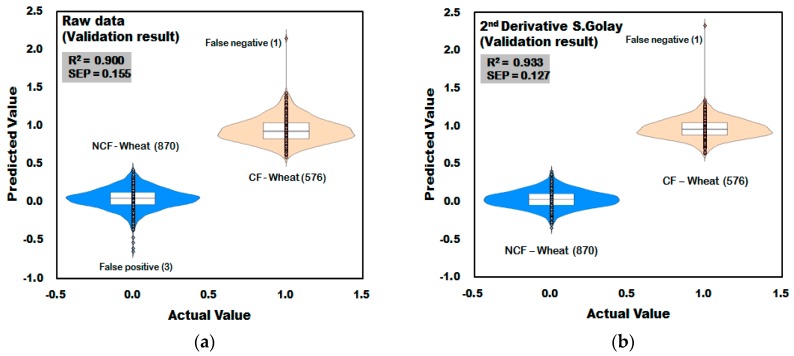
Validation results for the *Fusarium* discrimination of the PLS-DA model developed using raw reflectance spectra (**a**) and the second-order derivative pretreatment (**b**) obtained from wheat.

Figure 15a shows the result of validation model of PLS-DA developed using raw spectra acquired from wheat. In the developed validation model, one of the 576 spectra acquired from CF-wheat samples was predicted as higher than 1.5, which is the reference value, resulting in a CCR of 99.83% (575/576), and one of the 870 spectra acquired from the NCF wheat samples was predicted as lower than −0.5, which is the reference value, resulting in a CCR of 99.66% (867/870). Figure 15b shows the validation result of the PLS-DA model developed by applying second derivative pretreatment to raw spectra acquired from wheat samples. One of the 576 spectra acquired from the wheat samples contaminated with *Fusarium* was predicted as higher than 1.5, which is the reference value, resulting in a CCR of 99.83% (575/576). The R_V_^2^ of the validation model applying the second-order derivative pretreatment was 0.933, which is higher than the result of the raw spectra, and the SEP decreased to 0.127, proving the effect of the spectra pretreatment. 

## 4. Conclusions and Outlook

A technology to discriminate hulled barley, naked barley, and wheat contaminated with *Fusarium* rapidly and non-destructively was developed using near infrared spectroscopy technology. The PLS-DA models that included a first-order derivative, second-order derivative, or third-order pretreatment yielded an improved predictive discrimination accuracy for the infected grain samples (>99.5%), compared with the method without a pretreatment. In particular, the naked barley discrimination accuracy was the best when compared with other grains. The PLS-DA model that was developed by applying derivative pretreatment to the acquired reflectance spectra was advantageous in the improvement of the correct classification rate. In our experiment, the use of reflectance spectra based on the NIRS technique has been proposed as an innovative and specific instrument for the discrimination of grains contaminated with fungus. The obtained results suggest the possible applications for discrimination of grains contaminated with other fungi similar to *Fusarium*. In the future, for the practical validation of the developed PLS-DA model, experiments to discriminate grains contaminated with *Fusarium* will be performed by adding grain samples produced from other regions or harvested in different years.

## Figures and Tables

**Table 1:** Pictures and quantity of hulled barley, naked barley, and wheat samples.

Grains	Geographic Locations	Group	Quantity	Pictures	
Hulled barley	Gyeongsangbuk-do (Yeongdeok-gun)	GB3-C *	135	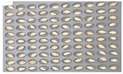	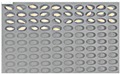
	Gyeongsangbuk-do (Gyeongju-si)	GB5	148	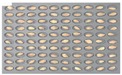	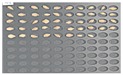
	Gyeongsangnam-do (Changnyeong-gun)	GN9	134	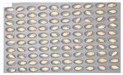	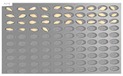
	Jeollabuk-do (Iksan-si)	JB11	159	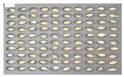	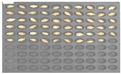
	Jeollabuk-do (Gimje-si)	JB9	149	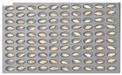	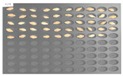
	Jeollanam-do (Yeonggwang-gun)	JN8	146	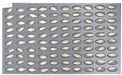	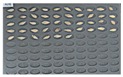
	Chungcheongnam-do (Taean-gun)	CN2	125	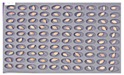	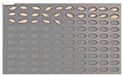
Naked barley	Gyeonggi-do (Hwaseong-si)	GG2-C *	163	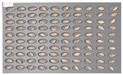	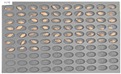
	Jeollanam-do (Hampyeong-gun)	JN13	200	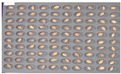	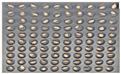
	Gyeongsangnam-do (Gimhae-si)	GN4	199	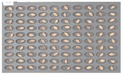	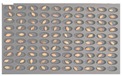
Wheat	Gangwon-do (Donghae-si)	GW1-C *	145	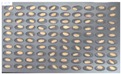	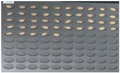
	Jeollabuk-do (Gimje-si)	JB11	125	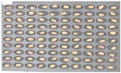	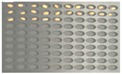
	Gyeongsangnam-do (Milyang-si)	GN2	123	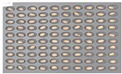	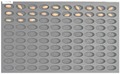

* Control group.

**Table 2:** Culture results for control group and experimental group of hulled barley, naked barley, and wheat samples contaminated with *Fusarium*.

Types of Grain		(a) Primary Culture Results (^1)^ NCF + ^2)^ CF = Sum)	(b) Secondary Culture Results (NCF + CF = Sum)
Hulled barley	GB3-C *	135 + 0 = 135	135 + 0 = 135
	GB5	147 + 1 = 148	147 + 1 = 148
	GN9	39 + 95 = 134	28 + 106 = 134
	JB11	158 + 1 = 159	159 + 0 = 159
	JB9	107 + 42 = 149	123 + 26 = 149
	JN8	117 + 29 = 146	131 + 15 = 146
	CN2	114 + 11 = 125	123 + 2 = 125
Naked barley	GG2-C *	163 + 0 = 163	149 + 14 = 163
	JN13	166 + 34 = 200	188 + 12 = 200
	GN4	172 + 27 = 199	178 + 21 = 199
Wheat	GW1-C *	145 + 0 = 145	145 + 0 = 145
	JB11	25 + 100 = 125	92 + 33 = 125
	GN2	73 + 50 = 123	60 + 63 = 123

^1)^ NCF: Not contaminated with *Fusarium*; ^2)^ CF: Contaminated with *Fusarium;* * Control group.

**Table 3:** Performance of the partial least square discrimination analysis (PLS-DA) calibration and validation models for *Fusarium*-contaminated hulled barley samples, as well as the classification accuracy for normal and contaminated hulled barley, naked barley, and wheat.

Pretreatment	^1)^ PC	Performances of Calibration				Performances of Validation			
		^2)^ R_c_^2^	^3)^ SEC	^4)^ CCR (%)		^5)^ R_v_^2^	^6)^ SEP	CCR (%)	
				NCF	CF			NCF	CF
***Hulled barley***									
Non-pretreatment	16	0.888	0.167	100	99.56	0.881	0.172	100	99.44
1st order Derivative	11	0.918	0.143	100	100	0.911	0.149	100	99.56
2nd order Derivative	10	0.950	0.112	100	99.78	0.946	0.116	100	99.78
3rd order Derivative	9	0.934	0.128	100	99.89	0.928	0.134	100	99.89
Mean ^7)^ Nor	13	0.863	0.185	98.52	99.00	0.855	0.190	98.52	98.89
Maximum Nor	13	0.855	0.190	98.52	99.00	0.846	0.196	98.15	98.78
Range Nor	14	0.869	0.181	98.89	99.11	0.860	0.187	98.77	99.00
^8)^ MSC	12	0.835	0.203	98.02	99.11	0.826	0.208	97.90	98.78
Baseline	15	0.875	0.177	100	99.44	0.868	0.182	99.88	99.22
^9)^ SNV	13	0.836	0.202	97.90	99.11	0.827	0.208	97.90	98.78
***Naked barley***									
Non-pretreatment	13	0.868	0.140	99.89	98.99	0.859	0.145	99.89	98.48
1st order Derivative	10	0.939	0.095	100	100	0.932	0.101	100	100
2nd order Derivative	7	0.944	0.091	100	100	0.941	0.093	100	100
3rd order Derivative	7	0.950	0.086	100	100	0.948	0.088	100	100
Mean Nor	12	0.810	0.168	99.22	98.99	0.800	0.174	99.22	98.99
Maximum Nor	13	0.810	0.170	99.22	98.99	0.792	0.176	99.22	98.99
Range Nor	13	0.807	0.169	99.22	98.99	0.792	0.176	99.22	98.99
MSC	11	0.777	0.182	99.33	98.99	0.758	0.190	98.99	98.48
Baseline	13	0.865	0.142	99.89	98.99	0.855	0.147	99.89	98.48
SNV	12	0.781	0.181	99.33	98.48	0.763	0.188	99.11	98.48
***Wheat***									
Non-pretreatment	12	0.908	0.149	99.66	100	0.900	0.155	99.66	99.83
First-order Derivative	8	0.923	0.136	100	100	0.909	0.147	100	99.83
Second-order Derivative	7	0.941	0.119	100	100	0.933	0.127	100	99.83
Third^-^order Derivative	5	0.916	0.141	100	99.83	0.911	0.146	100	99.83
Mean Nor	9	0.939	0.121	99.89	99.83	0.934	0.126	99.89	99.83
Maximum Nor	10	0.933	0.127	99.89	99.83	0.928	0.132	99.89	99.83
Range Nor	10	0.928	0.131	99.54	100	0.921	0.138	99.54	99.83
MSC	9	0.912	0.145	99.54	100	0.906	0.150	99.54	99.65
Baseline	13	0.909	0.147	99.89	100	0.901	0.154	99.89	99.65
SNV	10	0.913	0.144	99.54	100	0.908	0.149	99.54	99.65

^1)^ PC: Principle component number; ^2)^ R_c_^2^: Coefficient of determination for calibration; ^3)^ SEC: Standard error of calibration; ^4)^ CCR: Correct classification rate; ^5)^ R_v_^2^: Coefficient of determination for validation; ^6)^ SEP: Standard error of prediction; ^7)^ Nor: Normalization; ^8)^ MSC: Mean scattering correction; ^9)^ SNV: Standard normal variate.
